# MicroRNA-655 suppresses cell proliferation and invasion in oral squamous cell carcinoma by directly targeting metadherin and regulating the PTEN/AKT pathway

**DOI:** 10.3892/mmr.2022.12897

**Published:** 2022-11-14

**Authors:** Qiang Wang, Longkun Lv, Yuan Li, Honghai Ji

Mol Med Rep 18: 3106–3114, 2018; DOI: 10.3892/mmr.2018.9292

Following the publication of this paper, it was drawn to the Editors’ attention by a concerned reader that the data in the centre panel shown for the cell invasion assays in Fig. 2C were strikingly similar to data appearing in different form in other articles by different authors. Owing to the fact that the contentious data in the above article had already been published elsewhere prior to its submission to *Molecular Medicine Reports*, the Editor has decided that this paper should be retracted from the Journal. The authors were asked for an explanation to account for these concerns, but the Editorial Office did not receive a reply. The Editor apologizes to the readership for any inconvenience caused.

